# A Smart Sensor-Based Measurement System for Advanced Bee Hive Monitoring †

**DOI:** 10.3390/s20092726

**Published:** 2020-05-10

**Authors:** Stefania Cecchi, Susanna Spinsante, Alessandro Terenzi, Simone Orcioni

**Affiliations:** Dipartimento di Ingegneria dell’Informazione, Università Politecnica delle Marche, 60131 Ancona, Italy; s.spinsante@univpm.it (S.S.); a.terenzi@univpm.it (A.T.); s.orcioni@univpm.it (S.O.)

**Keywords:** beehives monitoring, real-time monitoring, weight measurement, sound measurement, temperature and humidity measurement

## Abstract

The widespread decline of honey bee (*Apis mellifera* L.) colonies registered in recent years has raised great attention to the need of gathering deeper knowledge about this phenomenon, by observing the colonies’ activity to identify possible causes, and design corresponding countermeasures. In fact, honey bees have well-known positive effects on both the environment and human life, and their preservation becomes critical not only for ecological reasons, but also for the social and economic development of rural communities. Smart sensor systems are being developed for real-time and long-term measurement of relevant parameters related to beehive conditions, such as the hive weight, sounds emitted by the bees, temperature, humidity, and CO${}_{2}$ inside the beehive, as well as weather conditions outside. This paper presents a multisensor platform designed to measure the aforementioned parameters from beehives deployed in the field, and shows how the fusion of different sensor measurements may provide insights on the status of the colony, its interaction with the surrounding environment, and the influence of climatic conditions.

## 1. Introduction

The role of honey bees (*Apis mellifera* L.) in the natural ecosystem and their importance for the health of the environment and life preservation are well known. These industrious insects do not only produce honey, beeswax, royal jelly, and propolis, but they are at the basis of the plants’ pollination process, playing a key role in the proliferation of both spontaneous and cultivated flora. For these reasons, it is of the utmost importance to design solutions aimed at preserving and supporting the honey bees’ healthy existence.

Colony collapse disorder (CCD) is a recent, pervasive syndrome affecting honey bee colonies in Europe and the rest of the world, which is characterized by a sudden disappearance of honey bees from the hive [1,2,3]. Causes of CCD are not perfectly clarified yet, but mites and viruses, malnutrition, pesticides, beekeeping practices, electromagnetic radiation, and genetically modified crops may be mentioned among the possible ones. The consensus among bee scientists is that the decline of honey bee colonies is the result of multiple stressors, acting independently, in combination, or synergistically to impact honey bee health [2,4,5]. CCD has emphasized the importance of enabling a continuous, multiparametric, and extensive monitoring of the beehives, to investigate factors that may negatively affect the life cycle of bees. Large bee mortality can result in loss of pollination services which has important negative ecological and economic impacts that could significantly affect the maintenance of wild plant diversity, wider ecosystem stability, and crop production. In this context, a continuous monitoring and an automatic analysis of the beehives’ status can help to safeguard and to protect their life, by early detection of potential threats, as also underlined in [6].

Focusing on a general monitoring system for beehives, some contributions can be found in the literature [6,7]. In [8], the results of the “Electronic Bee Hive” project are reported. In particular, the analysis of numerous sensors located at specific points inside the beehive, and capable of recording various parameters such as temperature, humidity level, carbon dioxide concentration, and also the weight of the beehive, has been considered. In [9], a method based on supervised machine learning approach that uses data from in-hive sensors (i.e., internal temperature and beehive weight), weather and apiary inspections to forecast the health status of honey bee colonies is presented. In [10], a web monitoring system based on internal sensors and a cloud architecture to monitor and follow bees’ behavior is described. In particular, temperature, humidity, air quality sensors, and an accelerometer compass are installed within the hive to collect the data that are managed through the proposed cloud architecture, realized with a lambda architecture and a scientific data sharing platform.

Focusing on the adoption of wireless technologies, several projects can be mentioned. In [11,12], a system composed of heterogeneous Wireless Sensor Networks (WSNs) technologies to gather data unobtrusively from a beehive has been described. In this case, a wide range of sensors were used such as oxygen, carbon dioxide, pollutant levels, temperature, and humidity measurement devices. In [13], a beehive monitoring system to monitor essential parameters of a beehive (such as temperature, sound, and weight) and additionally including an image recognition algorithm to observe the degree of infestation with Varroa mites has been reported. In [14], a remote monitoring system (called WBee) based on a hierarchical three-level model formed by the wireless node, a local data server, and a cloud data server, capable of measuring temperature, humidity inside a beehive, and its weight has been presented. A wireless sensor network system has been presented also in [15].

Systems exploiting computer vision-based approaches have been proposed too, to analyse the behaviour of honey bees and their flight activities outside the hive. In [16], a model to track bees in a 50 Hz frame rate 2D video of the hive entrance close view is proposed, which can track 99% of single flying bees and 70% of merged bees, in total. The proposed method, based on bees’ blobs segmentation, suffers a number of limitations associated to natural colors variation, and to the assumption of bees flying at a constant velocity. An improvement of this method has been proposed in [17,18]. Digital video cameras have been used to track the social interactions among bees also in [19]. The proposed solution is able to track up to 16 bees walking freely on a flat surface, with more than 95% of the individuals’ locations correctly extracted and traced. The prototype system works offline, on pre-recorded videos, and is able to monitor the bees’ behaviour within a very limited area, so it is more suitable for analysing specific motion patterns outside the hive, than for a long-term monitoring of the bees’ health status. In [20], a system for monitoring and analyzing the in-and-out activity of honey bees, as they pass through the entrance of a beehive, is proposed. It is based on an infrared system and a circular character-encoding: tags were attached to the dorsum of the bees’ thoraxes to identify the bee workers individually. The analysis is performed by a support vector machine (SVM) classifier and it allows ~98% and ~86% of identification accuracy rate. In [21], a real-time imaging system for multiple honey bees tracking and activity monitoring, by counting the honey bees entering and exiting the beehive, is presented. The system is based on the use of integrated Kalman Filter and Hungarian algorithm tracking method, and it is capable of determining the incoming and outgoing activity of individual honey bees with an automatic counting accuracy of 93.9% in comparison to manual counting.

Starting from the state of the art related to monitoring systems and technological developments, this work extends the results presented in [22] and aims at developing a complete, multiparametric smart sensor-based measurement system capable of monitoring in real-time a colony of beehives, and discriminating several events, such as swarming event, beehive theft, honey gathering, reserve food lack, decrease of bees’ number due to illness. Together with a detailed description of the system implementation and sensors installation, several experimental results obtained in a real scenario will be reported. With respect to the system presented in [23,24], a weight measurement system has been added and described in details in this paper, presenting additional results in terms of measured parameters.

In particular, Section 2 will describe the use of the sensors considered in our platform (such as sound, weight, humidity, temperature, and CO${}_{2}$) as referenced in the literature. Section 3 will report the presented measurement system considering for each sensor, hardware design, software implementation, and its final installation in a field deployment within our university campus. Section 4 will describe the obtained results taking into consideration several aspects, like seasonal patterns, swarming, and honey gathering. Finally, Section 5 will report conclusions and a future perspective.

## 2. State of the Art of Measurement Systems for Beehives

### 2.1. Weight Measurement

The analysis of the literature shows that among the different quantities of interest in beehive monitoring, the time variation of their weight can accurately reflect the productivity of the colony, as well as its health and well-being conditions. As a consequence, beehive weight measurement systems have great importance, especially the automatic solutions based on sensor networks that enable a continuous collection of data, and the early detection of upcoming negative conditions requiring a prompt intervention on the beehive.

Methods to measure the beehives’ weight usually adopted by beekeepers are generally very simple, but also inaccurate. In most cases, a basic tension scale is placed under the hive that is tilted on one side, assuming that weight in the hive is more or less centrally distributed. By doubling the available reading, the weight measurement value is obtained. Several weight-related figures may provide an accurate picture of the hive status, such as the diurnal variation of the hive weight, or the time profile of daily gains and losses, which are also useful to know in order to select the best time for harvesting the honey stores and optimize the hive management procedures. Gathering the above mentioned information through measurement operations requires the capability to weigh several tens of kilograms (typical order of magnitude of a beehive’s weight), but with an accuracy of tens of grams, which makes hive weight measurement a non trivial task. In fact, a measurement resolution of only 100 g would be inadequate for monitoring colonies during the passive state (winter period), when nectar is not harvested and the daily changes in mass do not exceed 100 g [25].

The manual weighing of hives, performed by the first experimenters looking for quantitative data to justify the trends in bee colonies’ decline, was based on the use of high precision mechanical scales suitably modified to be located under the hives. However, manual measurement reading with the required frequency is too laborious to perform at a large scale and in the long term. As a consequence, later approaches tried to develop automatic solutions for weight measurement, for which the use of load cells has emerged as the most common choice.

The design of a proper hive’s weight measurement scale for an automatic system has to take into account several requirements and constraints. In order to obtain a reliable measurement tool, the weight of the beehive shall be fully translated to the load cell, which usually requires the use of an interposed custom frame, designed according to the physical dimensions of the hive. Such a frame, typically an aluminum one, shall ensure uniform weight distribution, physical dimensions stability, being insensitive to environmental conditions such as relative humidity and temperature variations which could cause deformations in untreated woody structures, and robustness to possible imbalances of the beehive. Furthermore, the bottom of the hive is not always closed, and in some cases, it is possible to have at the bottom of the hive an iron net to ensure the air circulation during the spring and summer seasons. For these reasons, load cells can be located under the hive’s corners, and configured in so-called Wheatstone bridge circuit, aiming for greater accuracy in terms of voltage output difference, proportional to the varying weight to measure.

Looking at the state-of-the-art literature about technological solutions to automatically gather measurements of the beehives’ weight, several approaches may be found, even quite far in time. The summary herein provided is limited to the last three years, in order to focus on the most recently proposed solutions based on technologies comparable to those used in this work.

A low-cost platform to monitor the bees’ health and the amount of honey produced by them was developed in 2018 by Seritan et al. [26]. The measured parameters include inner and outer temperatures, humidity and weight, and the CO${}_{2}$ concentration inside the hive, which is representative of the bees’ health. An Arduino Nano hardware unit is employed to collect and locally process the data generated by a single load cell (TAL220 model (Sparkfun Electronics, Niwot, CO, USA) [27]) placed under the hive and used as the weight sensor. The selected load cell, an aluminum-alloy straight bar, includes four strain gauges and two precision resistors in a Wheatstone bridge configuration, powered at $V_{IN}$ = 5 V. The output voltage depends on the resistance values of the strain gauges, which can be properly selected to attain the desired accuracy in the measurement. In order to come up with a measurable signal, the conditioning circuit includes an HX711 load cell amplifier integrated circuit [28], providing a 24-bit precision analog-to-digital converter (ADC) that outputs the analog voltage as two-decimals converted digital values.

In [29], a system to detect swarming is presented, which exploits two different quantities, i.e., sound generated by bees during swarming and weight of the hive, which obviously changes whenever bees leave the hive. The authors mention the use of a load cell interfaced to an Arduino board to measure the weight of the hive, but they do not provide additional details about the specific hardware used and the attained accuracy in measurements. Focusing on the detection of the swarming events, it is observed that prior to the take-off of the bees, there seems to be a rapid rise in temperature and humidity moments before the observed drop in weight. The weight measurements of the hives were remotely gathered by using the electronic system named Wbee [14], in the experiments by Flores et al. [30]: the research focused on a flowering period in the beekeeping seasons of 2016 and 2017, marked by extreme episodes of drought and high temperatures. The weight measurement system consists of a scale used as a base for the beehives, and composed by a metallic frame (50 cm × 40 cm) with a 150 kg load cell associated, connected to a display. Based on the authors’ description of the set-up, the scale periodically sends the measurements over a serial link, with a programmed resolution of 100 g. The data analysis performed by the authors shows a relation between the hives’ weight and the amount of rain during the season, which can strongly affect the life of the colony.

The aim of the work presented by Shaghaghi et al. in [31] is to enable beekeepers getting specific and accurate data about which frames inside a hive are ready for harvesting, thus avoiding the costly and time-consuming activity of daily inspection of every frame within each hive of every apiary. In this work, the weight measurement of each frame is performed by using a pair of Force Sensing Resistors (FSRs), located at each ledge onto which the frame rests. The pressure at the intersection between a frame lug and a hivebox ledge, caused by the weight of the frame, is measured, and as the weight is distributed throughout the frame, the readings from each sensor at each end of the frame must be added together to calculate the frame weight. As expected, the use of FSRs exhibits several limitations, not only due to physical constraints in their correct placement, but mostly because of their time variant behavior due to aging effects and the lack of calibration, despite the data normalization process applied to increase measurements accuracy. As the authors state, FSRs cannot be reliably used to measure weight variations, and their replacement with different types of sensors, such as custom capacity-based ones, is finally foreseen.

Ochoa et al. [32] address the design of a multisensory measurement platform for precision beekeeping, aimed at gathering humidity, temperature, and weight measurements data to enable an optimized management of the hives, minimizing resources consumption and maximizing the productivity of the colony. As the hive weight may vary from 50 kg to 200 kg, four 50 kg load cells are used in conjunction with the HX711 Load Cell Transmitter (Avia Semiconductor, Xiamen, P.R. China): a 3D printed element facilitates their application onto the hive, even if the authors do not give details about their physical installation. The system data logger and the type of visual information provided to beekeepers are described in the paper, but no details are given about the accuracy of the weight measurements. Four strain gauge load cell sensors are used for weight measurement and integrated with the HX711 module in [33] too. They are installed at each edge under the hive topping; the authors state a ±0.1 kg accuracy for weight measurement. An autonomous beekeeping system is presented in [34]; the hive weight is measured by a specially-designed scale, and raw measurement data are sent by a sensor–transmitter to the gateway (or data concentrator) without additional processing. No details are however provided about the specific hardware components of the scale, and the accuracy of the attained measurements. The design of a smart monitoring system for stingless hives is given in [35]: four 20 kg load cells are placed per hive at each side corner, for a maximum 80 kg total weight to be measured. Again, the HX711 24-bit ADC is used. The authors report inaccurate weight readings, due to cells’ sensitivity and nonlinearity.

Strain gauges and the INA333 instrumentation precision amplifier (Texas Instruments, Dallas, TX, USA) [36] are used to measure weight in the embedded data acquisition (DAQ) and monitoring system presented in [37], but no more details are provided about the sensor components chosen.

The use of WSNs allows the long-range transmission of the data acquired locally by beehives’ monitoring systems, thus enabling the collection of measurement values in the long-term, and its processing in almost real-time at back-end servers. A WSN-based solution to continuously acquire environmental temperature, humidity, and hive’s weight measurements is presented in [38], with the aim of the automatic monitoring of the nectar flow within the colony. Environmental measurements and beehive’s data are collected by an Arduino Mega 2560-based end device that transmits the data to a ZigBee module operating at 2.4 GHz, which acts as the WSN coordinator. As seen in other solutions, a load cell located under the hive is used to measure in real time the hive’s weight, with a span of [0 ÷ 100] kg and an output sensitivity of the signal amplifier equal to (0.9 ± 0.2) mV/V. No details, however, are provided by the authors about the physical set-up of the load cell within the hive. The measurement data collected by the system were exploited to highlight variations of the beehive’s weight related to weather conditions, and the role played by the environmental variables in determining a reduction in honey production.

The design of a smart scale for beehive monitoring is presented in [39], which integrates a single point impact AMS-750 load cell modified to extend its load span. This way, a rated loading of 750 kg with an accuracy of 0.02% FSO (Full Scale Output) is achieved. The theoretical resolution enabled by an ultra-accurate 24-bit ADC (Analog Devices AD7190) is 11.4 g, well below the 100 g requirement: in fact, by removing noise and averaging the ADC output, an effective resolution in the range of tens of grams is obtained. The designed scale is fitted to the bottom of the brood chamber in the hive, fixing the load cell to two spacer blocks and two aluminum plates (base and platform). The measurement system is finally equipped with a low-power ZigBee wireless interface to transmit the collected data to a remote platform.

### 2.2. Sound Measurement

The sound analysis of the beehives is a useful technique applied to determine the bees’ state in a noninvasive manner [40].

The bees communicate each other using vibration and sound signals generated in several ways [41,42], such as gross body movements, wing movements, high-frequency muscle contractions without wing movements, and pressing the thorax against the substrates or another bee [43,44,45]. These signals are also strictly related to particular events, such as swarming and queen behavior during swarming [43,45] as we will see in what follows.

The sound can be recorded by means of microphones placed in specific position inside or outside the hives. As an alternative to the use of microphones, accelerometers sensors could also be used to sample the hive vibrations [46]. In relation to the beehives’s events, the correlation between the swarming prevision and the sound generated by the bees has been proven in [47,48,49,50,51]. In particular, the sound have been analyzed in the frequency domain by means of the Fourier transform before and after the swarming event and changes in terms of frequencies and amplitudes have been found. During the swarming event, it is also possible to retrieve from the audio analysis the presence of the queen bee. As a matter of fact, young queens emit piping sounds during the swarming process. These signals are directly transmitted to the substrate through close contact between the vibrating thorax and the comb [43,44]. These sounds can be identified through a frequency analysis and since they are characterized by a specific frequency, they allows to detect the presence of young queen inside the hive [43,44,52,53]. Moreover, during the year, also the worker bees produce different piping sounds with relation to the presence or the absence of the queen in the colony [45].

A continuous monitoring and analysis of the beehives’ sound could be used to analyze the presence of the queen bee during the normal activities of the hives. In the literature, it is possible to find alternative methods to detect the presence of a queen bee in a hive employing Radio Frequency Identification (RFID) tags placed on the bee body [54]. However, these techniques are more intrusive and time consuming compared to the sound analysis, as it is necessary to extract the queen bee from the hives and to apply the tag directly on its body. Furthermore, the use of microphones or accelerometers allows getting more detailed information on the general state of the hive, whereas using the tag method it is only possible to detect the presence of the queen. Another important event that can be derived from the sound analysis is the presence of airborne toxics in the hive as reported in [55]. This system is based on a profiling of the acoustic signatures of free-flying honey bee colonies, analyzing the resulting acoustic sounds to identify anomalies with relation to the specific properties of those acoustic sounds. All these studies demonstrate that the sound analysis provides a lot of data that can be processed to derive a more complete analysis of the state of health of the hive.

Recently, the use of Deep Learning and Machine Learning has been introduced to classify the recorded audio samples and to define an objective status of the analyzed beehives [56,57,58]. More results could be achieved in the next years as long as bigger audio datasets are collected from different research projects.

### 2.3. Humidity and Temperature Measurement

The analysis of the temperature and humidity inside and outside the hive can be helpful to understand some aspects of the colony. They are important parameters that can influence the bees’ health, the brood, and the productivity of the beehive. For example, several studies have shown that correct values of temperature and humidity can significantly decrease the mortality rate in the colony and they can increase the honey production. Focusing on the temperature measurement, it has been found that the temperature influences the bees and brood health and also that the productivity of the beehive is strongly affected by external ambient and internal hive conditions as discussed in [59]. As reported in [60,61,62], a correct temperature inside the hive can generate a significant decrease in the mortality rate of the colony and, at same time, it can increase honey production by reducing the internal consumption. In fact, it has been shown that bees consume honey to rise the internal temperature of the hive during the winter [63]. In [64], various system architectures implemented with different methods and approaches to monitor the beehive temperature were discussed. In [65], a solution for honey bee colony state identification using temperature data and fuzzy logic was proposed. The system is based on a fuzzy inference system that starting from the temperature data defines three possible states for the hive health status, i.e., normal, death, and extreme.

Relative Humidity (RH) is another important physical parameter that affects colony development and bees’ behavior, as shown in [66]. In [66,67,68], several studies capable of identifying and measuring the optimum range of humidity within the hive have been reported.

### 2.4. CO_2_ Measurement

The measurement of carbon dioxide (CO${}_{2}$) plays an important role for the analysis of the beehive behavior. In particular, it is linked to the bees’ metabolism, as a change in the respiratory emission of CO${}_{2}$ is associated to metabolic heating of a bee in its normal activity. Furthermore, when the carbon dioxide within the hive reaches much higher levels than the normal atmospheric ones, honey bees start using fanning and gas exchange events to expel the CO${}_{2}$-rich air, and to keep the CO${}_{2}$ at an acceptable level (i.e., between 0.1% and 4.3%) [69,70,71]. This means that this parameter is also related to the internal humidity and temperature and the quantity of sound generated by the bees, which can vary with fanning and gas exchange events.

## 3. Hardware and Software Implementation of the Multiparametric Measurement System

A multiparametric acquisition platform has been developed in order to acquire weight measurements, the sound generated by the honey bees, the temperature, the humidity, and also the amount of CO${}_{2}$ generated within each hive.

**Figure 1:**
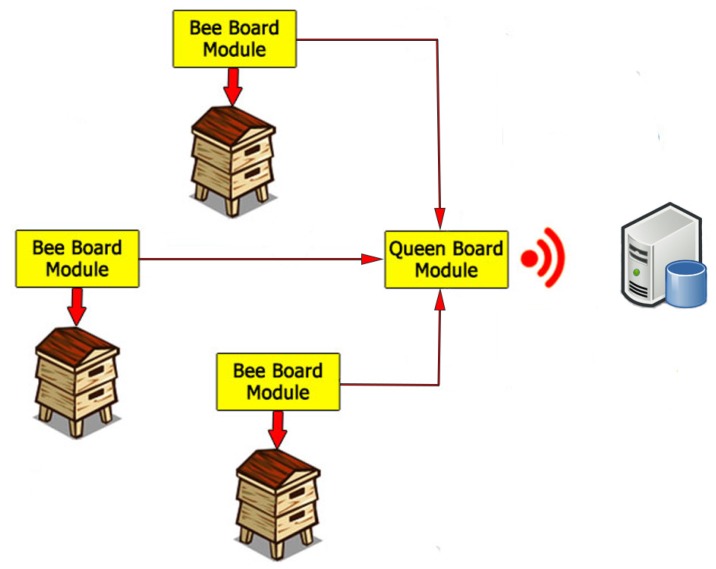
Connection between the Bee board and the Queen board modules with the remote server.

As shown in Figure 1, the system is modular and it is composed of two main modules. The former, named Bee board, is installed in each hive, and the latter, named Queen board, is the main module installed near the colony and it is capable of gathering the data from all the Bee board modules installed in each hive. The Bee board module consists of a RaspberryPi 3B equipped with a Behringer UCA22 sound card (MusicTribe, Manchester, UK), two ADMP401 MEMS microphones (Analog Devices, Norwood, MA, USA), two DHT22 humidity and temperature sensors (Aosong Electronics, Guangzhou, P.R. China), a properly designed weight scale, and a CO${}_{2}$ sensor. The Queen board module consists of a RaspberryPi 3B equipped with several sensors to acquire weather parameters near the hives and an Ethernet switch that, through a 5 GHz wireless bridge, allows the communication to a remote server where the acquired data is verified and stored. The system has been installed in June 2017 and it acquires data continuously. Regarding the power supply, the system is currently connected to the main power system of the university. However, the power consumption for the Bee board module is ~4.2 W while the Queen module has a consumption of about 4 W, allowing the use of a portable power supply or a properly designed energy harvesting solution.

### 3.1. Weight Measurement Sub-System

**Figure 2:**
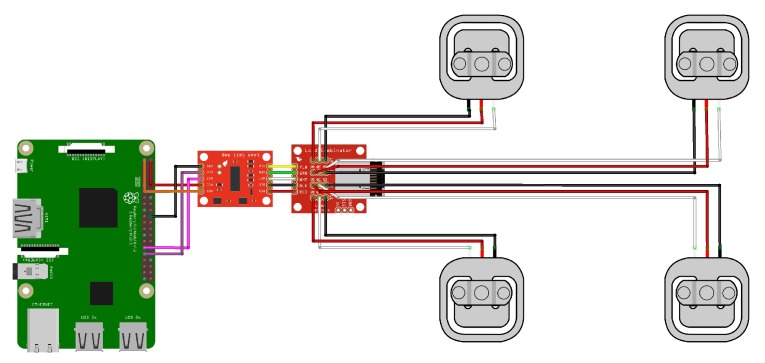
Schematic of electrical connections for the hive weight measurement system: the four load cells are connected in a Wheatstone bridge configuration, and the resulting signal is amplified before being acquired by the Raspberry Pi board.

**Figure 3:**
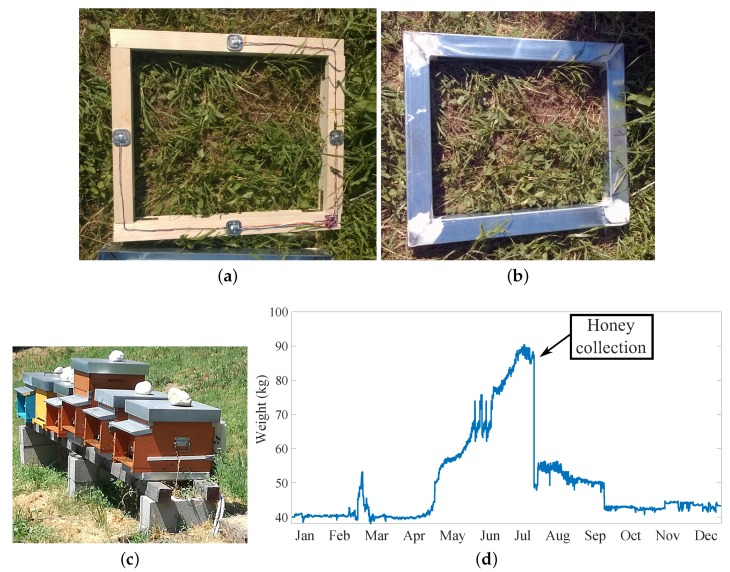
Implementation of the weight measurement system: (**a**) load cells installation, (**b**) aluminum covering frame, (**c**) weight measurement system installation under each hive within the bee colony, positioned in the University campus, and (**d**) weight measurements collected during 1 year.

The system used to measure the hive’s weight includes four load cells connected in a Wheatstone bridge configuration. The strain gauge Sparkfun load sensors [72] with a nominal capacity of 50 kg are chosen and connected to the RaspberryPi as shown in Figure 2. A Sparkfun load combinator PCB wires the four load cells together, and is connected to the HX711 load cell amplifier [28], the same as that used in the work recently published by Catania and Vallone [73]. The latter is a 24-bit ADC specific for weight scale applications, which also provides the sensors’ excitation voltage. The signal acquired from the bridge is first amplified and then sampled: the obtained values are finally transferred to the Raspberry Pi by means of a serial communication protocol. In Figure 3, the whole process of the load cells weight measurement sub-system installation is shown. First, the load cells are fixed at the middle of each side of a rectangular wood frame, having the same physical dimensions of the beehive support, displayed in Figure 3a. Then, an aluminum covering frame is applied on top the wood support, to protect the load cells from environmental effects, as shown in Figure 3b. Finally, Figure 3c shows the supports positioned under each hive. The same picture shows the entire colony, including several hives, located within the University campus.

**Figure 4:**
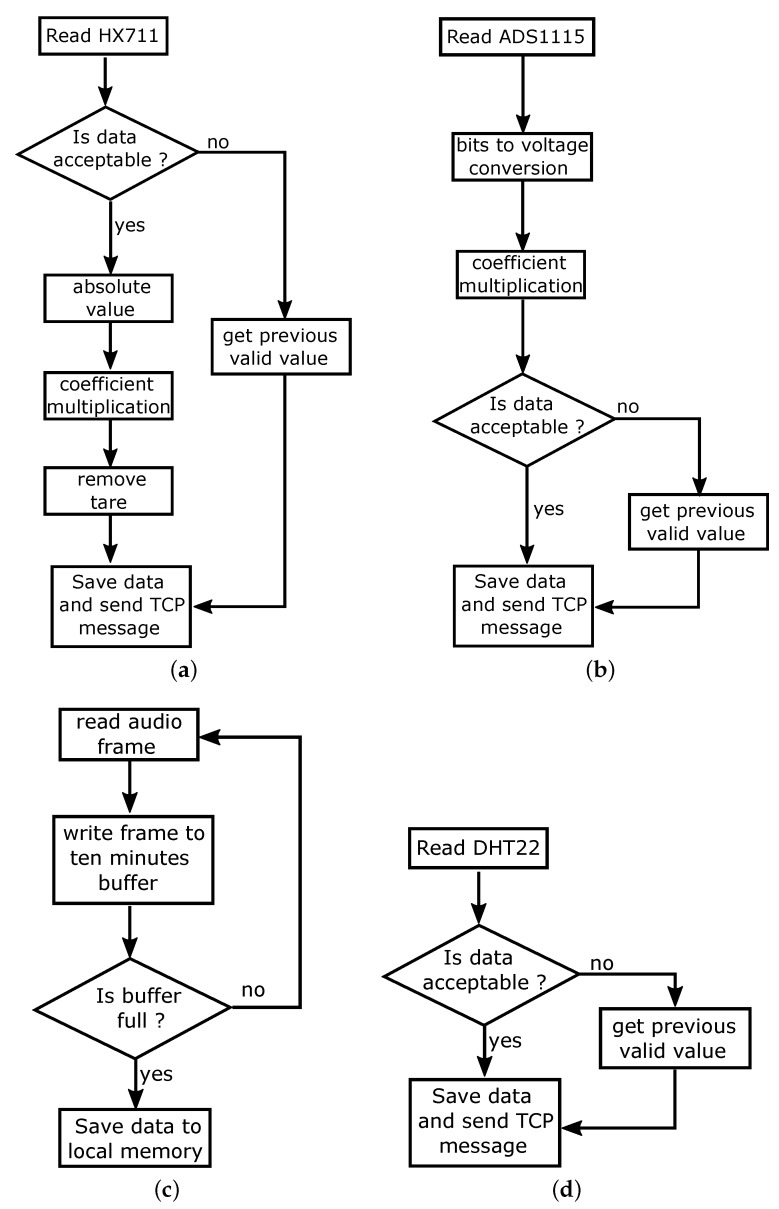
Flow charts of data acquisition routines, considering (**a**) weight acquisition code, (**b**) CO${}_{2}$ acquisition code, (**c**) temperature and humidity code, and (**d**) sound acquisition code.

The software component of the weight measurement sub-system is developed as a python script running on the Bee board module. A digital value is taken from the HX711 converter every 5 s, corresponding to a sample frequency of 0.2 Hz, by calling a function of the pigpio library [74]. The flow diagram in Figure 4a shows how each raw value is processed.

First, a check on the integrity of the incoming data is performed, to verify the correct reading from the sensor. In the case of a faulty reading (i.e., a NONE variable type is received from the sensor), the previous valid one is used to replace the missing new value. In the case of a successful reading, the absolute value of the new data is taken and multiplied by the weight scale constant k=0.04 to obtain the weight measurement at time *t*, namely W(t). The weight scale constant *k* is determined by a calibration process of the weight scale. After subtracting the tare value from the obtained weight measurement W(t), the data is finally ready for real time visualization and saving.

The hive’s weight measurement sub-system is tested and calibrated before being deployed in the field. The calibration process of the four load cells system allows to determine the scale constant *k*, used to convert the HX711 output raw values into weight measurements. To this aim, several different known weights are measured by using the developed system, then a proportionality coefficient between the raw values and the real weights is determined, getting to an estimated scale constant k=0.04. The three scales realized for the three hives have the same *k* value. Finally, the uncertainty is evaluated according to [75]: a known weight of 500 g is put on the scale and a vector *X* of 100 consecutive measurements is recorded. The uncertainty δx is estimated by Equation (1):

(1) $$\delta x=\frac{max(X)-min(X)}{2}$$

providing δx=±10 g. Finally the tare of each scale is determined too.

Figure 3d shows as an example the weight measurements collected during one year of acquisition: the increase of the hive’s weight during the spring and summer seasons, leading to almost a double value in July with respect to January, is clearly visible.

### 3.2. Sound Measurement

**Figure 5:**
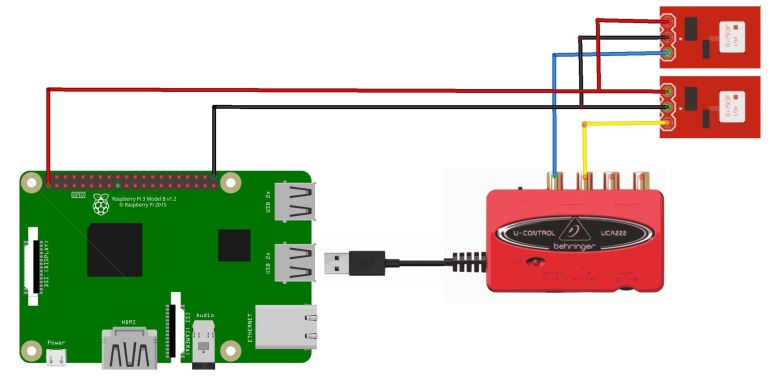
Schematic of electrical connections for the sound acquisition. The signal of ADMP401 microphone is acquired by means of Behringer UCA222 USB audio card which is connected to the Raspberry Pi board.

For sound measurement, two microphones are installed in each wood hive in two different positions. The former is in the center position of the front side of the hive, while the latter is in the center of the back side, housed in a dug into the wood. In order to have a full integration of the system, MEMS (Micro Electro-Mechanical Systems) microphones have been chosen. This way it is possible to keep the whole system as hidden and smaller as possible. The selected microphone is the Analog Devices ADMP401 (Analog Devices, Norwood, MA, USA) [76] with a frequency bandwidth between 100 Hz and 15 kHz. The microphones signals are acquired by a 24 bit USB sound card connected to the Raspberry Pi (Raspberry Pi Foundation, UK) with a sample rate of 32 kHz. Electrical connections of the system are visible in Figure 5. Each microphone has its own board that is fed with a 3.3 V power supply obtained by the Raspberry Pi GPIO. The analog signal is obtained from the USB soundcard. The advanced Linux sound architecture (ALSA) driver have been used with pyaduio library to develop the acquistion procedure. Figure 4d shows the acquisition routine: each frames is saved inside an acquisition buffer which is saved as a .wav file every ten minutes.

### 3.3. Humidity and Temperature Measurement

The relative humidity (RH) and temperature measurements have been performed using the DHT22 sensor [77], a low cost and small one-wire digital sensor. Three DHT22 sensors have been considered for each hive, placing two of them inside the hive for data acquisition, and one outside the hive and close to the electronic board, to monitor possible system overheating. In particular, the sensors inside the hive have been positioned in the center of lateral side and in the center of the back side, directly installed in the wood panels.The sensor device has the following characteristics:

- a measurement span from −40 °C to +80 °C, with an accuracy of ± 0.5 °C and a resolution of 0.1 °C for the temperature;
- a measurement span [0÷100]% RH with an accuracy of ±2% RH, and a resolution of ±0.1% RH, for the humidity.

**Figure 6:**
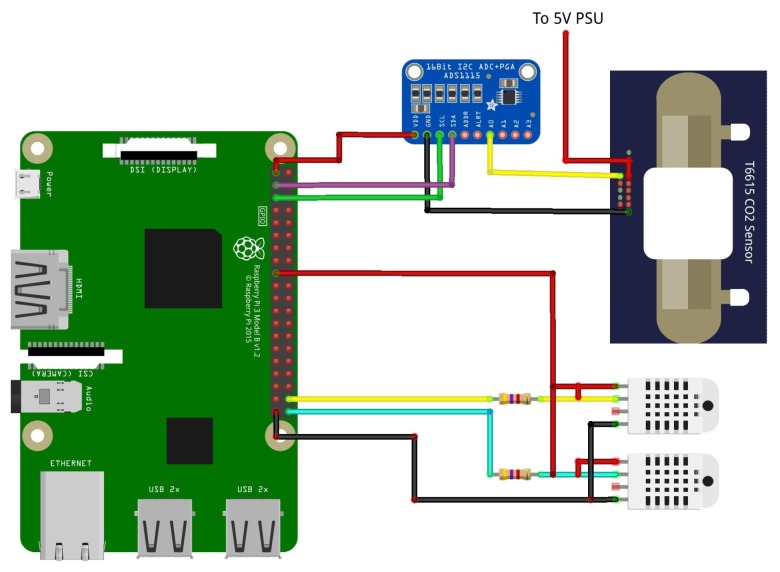
Schematic of electrical connections for T6615 CO${}_{2}$ sensor and the DHT22 temperature/ humidity sensors. The carbon dioxide sensor is powered by an external power supply unit since it has a current consumption which is not manageable from the Raspberry Pi GPIO. The output of the CO${}_{2}$ sensor is an analog signal which is acquired by the ADS 1115 component.

Electrical connections for DHT22 sensors are visible in Figure 6.

**Figure 7:**
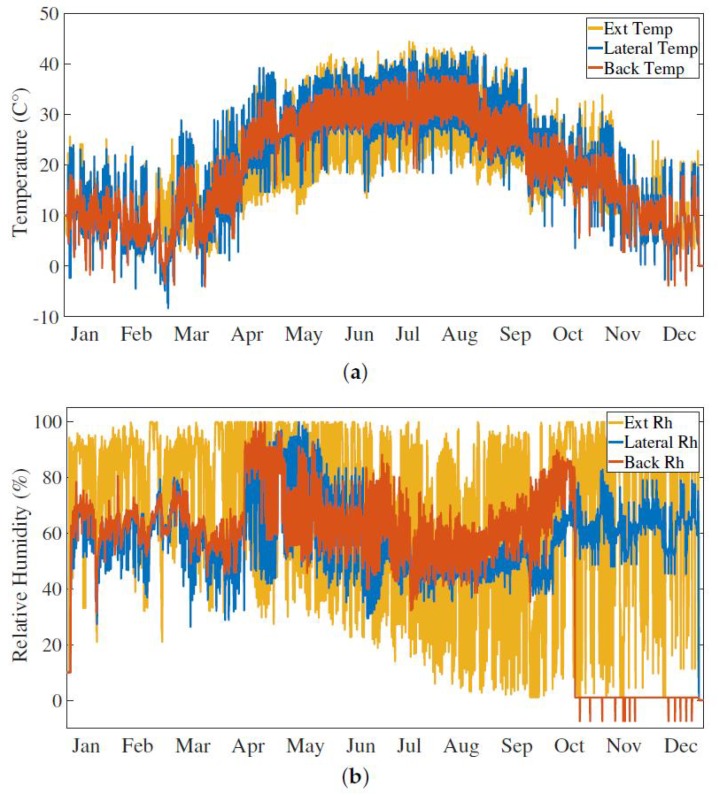
(**a**) Temperature and (**b**) relative humidity measurements during one year: in both cases, the first sensor is positioned on the inner lateral side of the hive (Lateral), the second sensor is positioned on the inner back side (Back), and the external sensor is positioned outside the hive (Ext).

Furthermore, according to sensor datasheet [77], a shielded cable has been used to connect the sensors to the Raspberry GPIO in order to avoid electrical interference which can degrade the measurement readings. Figure 4c shows the code acquisition routine. DHT22 sensor is a cheap sensor and it is well known for its occasional spurious readings; for this reason each acquired value is checked in order to verify its validity. If the value is not acceptable, the data is rejected and the previous value is kept as the valid one. Figure 7 shows an example of temperature and relative humidity measurement data acquired over one year. In Figure 7a, the concordance among the temperature values recorder by the three sensors is clearly visible, meaning that the inner thermal behavior of the hive follows the outer trend, and no overheating of the system is detected. On the other hand, despite the missing data during months October to December due to the faulty back RH sensor, Figure 7b shows a less strong correlation among the outer and inner relative humidity values. As expected, the inner sensors’ readings highlight the same trend of the relative humidity: much less variable than the external one. Bees are able to keep the inner relative humidity quite stable during the year.

### 3.4. CO_2_ Measurement

**Figure 8:**
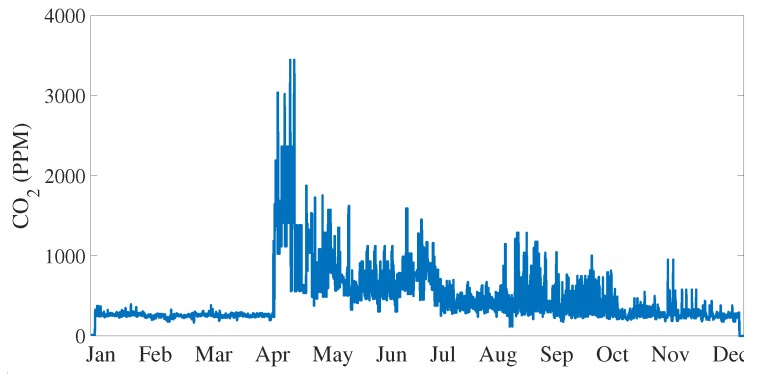
CO${}_{2}$ measurement during one year.

The carbon dioxide $CO_{2}$ measurement has been performed using the Telaire TL6615 sensor (Amphenol Advanced Sensors, St. Marys, NJ, USA) [78]. It is realized in Non-Dispersive Infrared (NDIR) flow-through technology and it can measure in the range [0 ÷ 50,000] ppm with an accuracy of 75 ppm on 10% of reading. During the measurement, the sensor generates an analog output from 0 V to 4 V, that is acquired by a Texas Instruments ADS115 (Texas Instruments, Dallas, TX, USA) which is an $I^{2}C$ Analog to Digital Converter (ADC) capable of 16-bit resolution and a sample rate of 860 Hz. The sensor has been installed directly in the wood panel and it is positioned in the lower part of the back side. Electrical connections are visible in Figure 6 in particular as the T6615 has an high current consumption, the sensor is feeded directly from the power supply unit and not through the Rapsberry GPIO. Figure 4b shows the acquisition routine to process CO${}_{2}$ readings. First, the raw data acquired from the ADS1115 ADC are converted from bit to voltage readings, then according to the datasheet [78], the voltage readings are converted into a CO${}_{2}$ PPM value. Moreover, in this case, the data acquired are checked in order to avoid false readings. Figure 8 shows the measurement values collected during one year of acquisition: the increased activity of bees is evident with the start of the spring season, when a spike in the emission of CO${}_{2}$ is registered.

## 4. Experimental Results

By analysing the measurement data acquired from the colony positioned within the campus, where the multisensor system was installed, different conditions and events related to the bees’ life and activities have been observed, namely,

- the normal activity of the beehives during a day;
- the honey gathering over one week comparing two seasons, i.e., spring and summer; and
- swarming event registered in two different hives.

**Figure 9:**
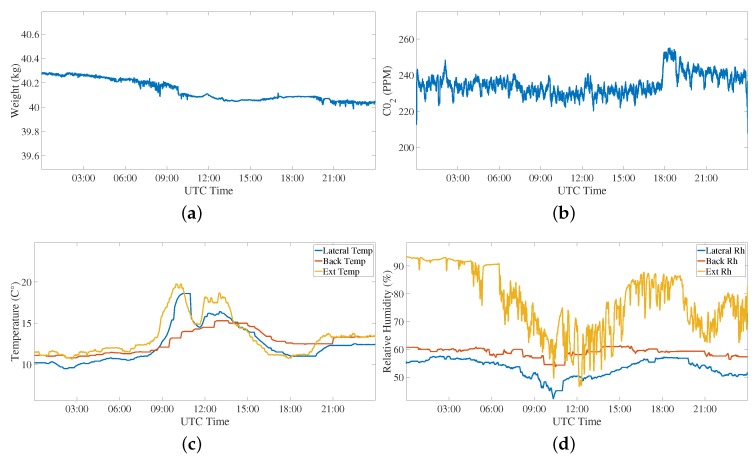
First event: normal activity of the bee hives monitored through the analysis of (**a**) weight, (**b**) CO_2_, (**c**) temperature, and (**d**) relative humidity variations measured over 24 h on 1 February 2018. During the winter, there is a small activity of the hive confirmed by the measured data.

**Figure 10:**
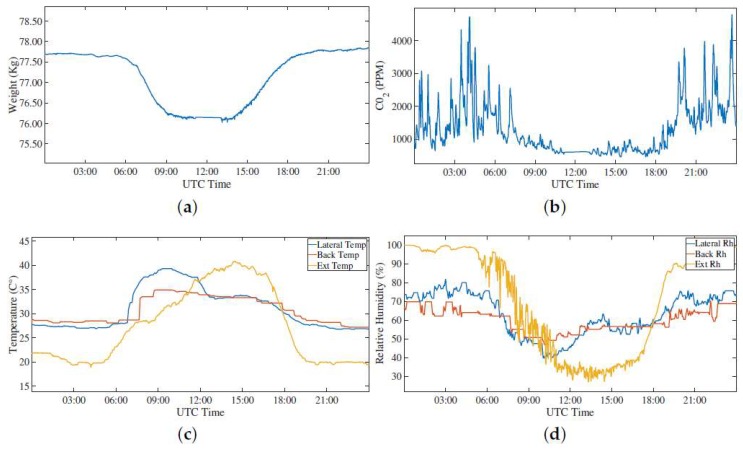
First event: normal activity of the bee hives monitored through the analysis of (**a**) weight, (**b**) CO_2_, (**c**) temperature, and (**d**) relative humidity variations measured over 24 h on 1 June 2018. During the spring, there is a large activity in the central part of the day confirmed by the measured data.

Regarding the first event, i.e., the normal activity of the hive, an entire day (24 h) in two different periods of the year has been considered, taking into consideration the measured weight, CO_2_, relative humidity, and temperature values. Figure 9 show the data from February (i.e., winter season) while Figure 10 shows the data from June (i.e., summer season). Taking into consideration the weight, it is evident from the comparison between Figure 9a and Figure 10a that during the summer days the honey bees go out from the hive to collect pollen and nectar, and they get back at night. During the winter season, basically the bees do not leave the hive, and the slowly decreasing weight shows that they are harvesting their store to feed themselves. We can see in Figure 10a a variation of 1 kg and a little increase at the end of the day giving the possibility to distinguish this event from others, like swarming. This fact is also confirmed by the CO_2_ measurement that shows a decrease during the same time interval of the weight variation. In relation to the temperature, there is an increase in the central part of the day, that is more evident in the summer when the bees go outside for the pollen. This aspect is related to a decrease of humidity during the same interval of the day, due to less bees remaining inside the hive.

**Figure 11:**
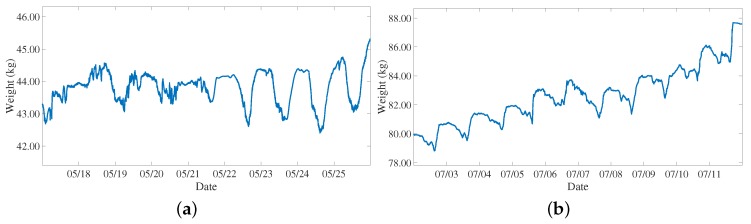
Second event: honey gathering derived from weight variations over one week: (**a**) spring season and (**b**) summer season.

Taking into consideration the second event, i.e., the honey gathering, the analysis has been focused on the weight measurement. Figure 11a shows the hive weight trend during the spring season, while Figure 11b shows the weight variation during the summer season considering a period of one week. In spring, the weight shows a variable pattern due to the fact that the honey is used for the bees nutrition, in the summer the weight is consistently increasing with a periodic pattern due to the honey gathering activity performed by the bees. This increasing oscillating characteristic of the weight is also reported in [79].

**Figure 12:**
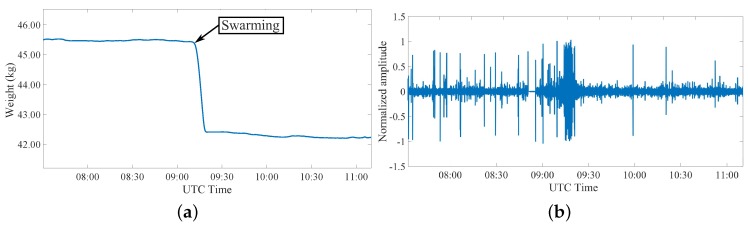
Third event: measurement during the swarming event in colony 1 during a day in April 2019 considering (**a**) weight variations and (**b**) related changes in recorded sound inside the colony.

**Figure 13:**
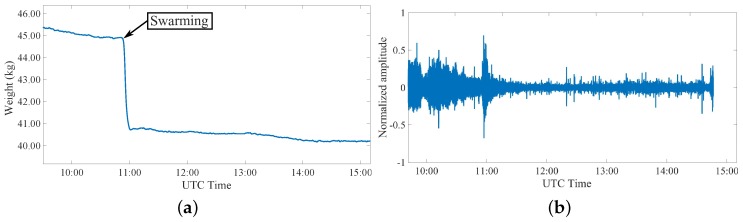
Third event: measurement during the swarming event in colony 2 during a day in June 2019 considering (**a**) weight variations and (**b**) related changes in recorded sound inside the colony.

The last analyzed phenomenon is the swarming event that starts during the spring as a consequence of an increased and strong population of the hive. A consistent part of this population decides to create a new group that moves away from the original hive. This event represents the natural way a honey bee colony uses to reproduce itself [49], and it becomes a positive occasion for beekeepers to increase the hives number in the colony, if there is the possibility to foresee this event and retain the new hive before it flies away. Two important characteristics of this event are the weight, that is surely modified, and the sound that increases during the swarming. Figure 12a and Figure 13a show the measured parameters during this event for two different colonies. It is evident in both cases a sudden weight variation between 3 and 4 kg that indicates a consistent part of the population of the hive is moving away. This fact has been correlated with the sound measurements, showing that the swarming event corresponds to an increase of the sound activity, as clearly visible in Figure 12b and Figure 13b.

## 5. Conclusions and Future Works

A multisensor platform capable of realizing a real-time and long-term measurement of relevant parameters related to beehives’ conditions, such as the hive weight, sounds emitted by the bees, temperature, humidity and CO_2_ inside the beehive, as well as weather conditions outside, has been presented. The system is composed of two modules, one installed on each hives of the colony (i.e., the Bee Board Module) and the other one handling all the data acquisitions, including also the weather parameters (i.e., the Queen Board module). Several analyses have been performed considering the measured parameters and some events that can occur within the colony. In particular, the normal activity of the bees during a day, the honey gathering over one week, comparing the spring and the summer season, and the swarming event registered in two different hives have been considered. From the results, it is evident that long term measurement of these parameters in real time plays a fundamental role in enabling advanced beehives monitoring, and the collected data can be used as a strong indicator of honey bees’ health. Future works will be oriented on the introduction of a server-side automatic tool for the determination of honey bees’ status taking advantage of advanced algorithms of digital signal processing.
